# NSUN5 Facilitates Hepatocellular Carcinoma Progression by Increasing SMAD3 Expression

**DOI:** 10.1002/advs.202404083

**Published:** 2024-11-12

**Authors:** Hexu Han, Chengcheng zhang, Wenbo Shi, Jiawei Wang, Wenhui Zhao, Yanping Du, Zhibin Zhao, Yifan Wang, Maosong Lin, Lei Qin, Xiaoxue Zhao, Qianqian Yin, Yiyi Liu, Zhongqi Wang, Jing Zhang, Xiaomin You, Guoxiong Zhou, Honghui Wu, Jun Ye, Xianzhong He, Weizhong Tian, Hong Yu, Yin Yuan, Qiang Wang

**Affiliations:** ^1^ Department of Gastroenterology The Affiliated Taizhou People's Hospital of Nanjing Medical University Taizhou School of Clinical Medicine Nanjing Medical University Taizhou Jiangsu 225300 P. R. China; ^2^ Department of Medical Oncology, Longhua Hospital Affiliated to Shanghai University of Traditional Chinese Medicine Shanghai University of Traditional Chinese Medicine 725 Wanpingnan Road Shanghai 200032 P. R. China; ^3^ Oncology major Ruijin‐Hainan Hospital Shanghai Jiao Tong University School of Medicine Hainan 200032 China; ^4^ Department of Hepatobiliary Surgery The Affiliated Taizhou People's Hospital of Nanjing Medical University Taizhou School of Clinical Medicine Nanjing Medical University Taizhou Jiangsu 225300 P. R. China; ^5^ Department of General Surgery First Affiliated Hospital of Suzhou University Suzhou 215000 P. R. China; ^6^ Department of Basic Medicine Jiangsu College of Nursing Huai'an Jiangsu 223001 P. R. China; ^7^ Department of Gastroenterology, Affiliated Hospital of Nantong University Nantong University Jiangsu 226001 P. R. China; ^8^ Reproduction Medicine Centre The Affiliated Taizhou People's Hospital of Nanjing Medical University Taizhou 225300 P. R. China; ^9^ Center for Translational Medicine, The Affiliated Taizhou People's Hospital of Nanjing Medical University, Taizhou School of Clinical Medicine Nanjing Medical University Taizhou Jiangsu 225300 P. R. China; ^10^ Department of Hepatobiliary Surgery The First Affiliated Hospital of Anhui Medical University Innovative Institute of Tumor Immunity and Medicine (ITIM) Anhui Provincial Innovation Institute for Pharmaceutical Basic Research Anhui Province Key Laboratory of Tumor Immune Microenvironment and Immunotherapy Hefei Anhui 230000 P. R. China; ^11^ Department of Radiology The Affiliated Taizhou People's Hospital of Nanjing Medical University Taizhou School of Clinical Medicine Nanjing Medical University Taizhou Jiangsu 225300 P. R. China; ^12^ Department of Pathology The Affiliated Taizhou People's Hospital of Nanjing Medical University Taizhou School of Clinical Medicine Nanjing Medical University Taizhou Jiangsu 225300 P. R. China

**Keywords:** EMT, hepatocellular carcinoma (HCC), metastasis, NSUN5, SMAD3

## Abstract

Hepatocellular carcinoma (HCC) is characterized by frequent intrahepatic and distant metastases, resulting in a poor prognosis for patients. Epithelial–mesenchymal transition (EMT) plays a pivotal role in this process. However, the expression of NOP2/Sun RNA methyltransferase 5 (NSUN5) in HCC and its role in mediating EMT remain poorly understood. In this study, clinicopathological analyses are conducted across multiple independent HCC cohorts and induced tumor formation in Nsun5‐knockout mice. The findings reveal an upregulation of NSUN5 expression in tumor tissues; conversely, the absence of Nsun5 hinders the malignant progression of HCC, indicating that NSUN5 may serve as a significant oncogene in HCC. Furthermore, elevated levels of NSUN5 enhance EMT processes within HCC cells. NSUN5‐knockout cells exhibit reduced invasion and migration capabilities under both in vivo and in vitro conditions, while overexpression of NSUN5 yields opposing effects. Mechanistically, high levels of NSUN5 promote the enrichment of trimethylated histone H3 at lysine 4 (H3K4me3) at the promoter region of SMAD3 through recruitment of the WDR5, thereby facilitating HCC metastasis via SMAD3‐mediated EMT pathways. Collectively, this study identifies NSUN5 as a novel driver of metastasis in HCC and provides a theoretical foundation for potential therapeutic strategies against this malignancy.

## Graphical abstract

This study identifies NSUN5 as a pivotal molecule that promotes the progression of HCC at multiple levels. Furthermore, through the application of various high‐throughput sequencing techniques, this research reveals that NSUN5, a classical RNA‐binding protein, is capable of binding to DNA and recruiting WDR5, a key epigenetic regulator. This interaction modulates the transcriptional regulation of SMAD3, thereby facilitating its biological functions through EMT.

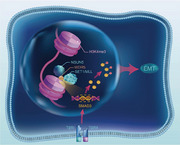

## 1 Introduction

Hepatocellular carcinoma (HCC) is the primary malignancy of the liver and is the predominant cancer type in China, with high global prevalence.^[^ ^1^ ^]^ Recently, methods for diagnosing and treating HCC have substantially improved.^[^ ^2^ ^]^ However, many patients still miss the opportunity for radical surgery because of early intrahepatic metastasis.^[^ ^3^ ^]^ Therefore, factors that facilitate metastasis should be further explored to improve early diagnosis rates.

Epithelial–mesenchymal transition (EMT) occurs when epithelial cells lose their basement membrane polarity, intercellular tight junctions, and adhesion under the influence of specific factors.^[^ ^4^ ^]^ This results in the transformation of these cells into invasive and migrating mesenchymal cells, contributing to tumor drug resistance.^[^ ^5^ ^]^ Typically, epithelial cells are closely interconnected and exhibit distinct polarity,^[^ ^6^ ^]^ characterized by structural and functional differences between their surfaces, with one end facing the luminal surface of the body or a body cavity.^[^ ^7^ ^]^ This polarity is maintained by intercellular junctions, including tight, intermediate, and gap junctions, as well as desmosomes, which play roles in cellular arrangement and interactions.^[^ ^8^ ^]^ Damage to intercellular junctions in epithelial cells disrupts cell polarity.^[^ ^9^ ^]^ Consequently, E‐cadherin expression decreases, whereas N‐cadherin and vimentin expression increases, promoting the invasiveness of tumor cells.^[^ ^10^ ^]^ Invasive tumor cells degrade the basement membrane, a crucial physical barrier that limits the expansion of the tumor cell mass.^[^ ^11^ ^]^ The resulting components trigger the release of cytokines that promote cell growth and survival—a process typically confined within the extracellular matrix under normal conditions.^[^ ^12^ ^]^ Recent studies have highlighted the critical role of EMT in initiating cancer metastasis.^[^ ^13^ ^]^ Therefore, identifying factors driving EMT in HCC is essential to enhance our understanding of the molecular pathological mechanisms of HCC and improve prevention and treatment strategies against distant HCC metastasis.

The biological process of EMT is directly regulated by various transcription factors, including ZEB1/2, TWIST, and Snail1/2.^[^ ^14^ ^]^ These transcription factors are regulated by multiple signaling pathways.^[^ ^15^ ^]^ Among these, the TGFB/SMAD3 pathway is a prominent cell signaling pathway that regulates numerous biological processes, including EMT.^[^ ^16^ ^]^ This signaling pathway is transduced as follows.^[^ ^17^ ^]^ First, transforming growth factor‐β (TGFB), a cytokine, binds to its receptor on the membrane of recipient cells, forming the TGFB receptor complex.^[^ ^18^ ^]^ Second, alongside the TGFB receptor complexes, SMAD3 is phosphorylated and subsequently activated, allowing its translocation into the nucleus where it participates in transcriptional regulation.^[^ ^19^ ^]^ Third, within the nucleus, phosphorylated and activated SMAD3 interacts with other transcription factors to regulate the expression of EMT‐related genes.^[^ ^20^ ^]^ However, the specific regulatory mechanisms of these crucial molecules and signaling pathways in HCC remain poorly understood.^[^ ^21^ ^]^ Therefore, further exploration of the factors regulating the TGFB/SMAD3 signaling pathway in HCC is necessary to establish a theoretical basis for developing preventive and therapeutic targets for HCC in the future.

NSUN5, an RNA methyltransferase, plays various roles under different physiological and pathological conditions.^[^ ^22^ ^]^ Schosserer et al. discovered that NSUN5 influences stress response and lifespan in fruit flies and worms by regulating ribosomal RNA methylation.^[^ ^23^ ^]^ NSUN5 is essential for the development of the cerebral cortex,^[^ ^24^ ^]^ and its deficiency in oligodendrocyte precursor cells is frequently observed in Williams–Beuren syndrome.^[^ ^25^ ^]^ Additionally, NSUN5 deficiency in gliomas leads to abnormal translation processes.^[^ ^26^ ^]^ Conversely, NSUN5 is highly expressed in colorectal cancer tumors, accelerating the cell cycle and promoting tumor proliferation.^[^ ^27^ ^]^ The varying levels of NSUN5 expression in different tumors reflect differences in the downstream mechanisms mediated by NSUN5 and its diverse functions in various diseases. However, the specific biological role of NSUN5 and its expression in HCC remains unclear. Currently, only one bioinformatics study by Zhang et al. in 2022 suggests that NSUN5 may be involved in the malignant process of HCC.^[^ ^28^ ^]^ However, the exact molecular mechanism remains unknown. This study investigated the role of NSUN5 in HCC using multicohort clinical samples combined with multiomics analysis and molecular experiments. The findings revealed that NSUN5 may act as a novel promoter of HCC metastasis. Mechanistically, highly expressed NSUN5 interacts with WDR5 to enhance the distribution of H3K4me3 in the SMAD3 promoter region, thereby promoting SMAD3 expression and enhancing the activation of this signaling pathway to facilitate EMT in HCC.

## 2 Results

### 2.1 NSUN5 is Highly Expressed in HCC Tumor Tissues

**Figure 1:**
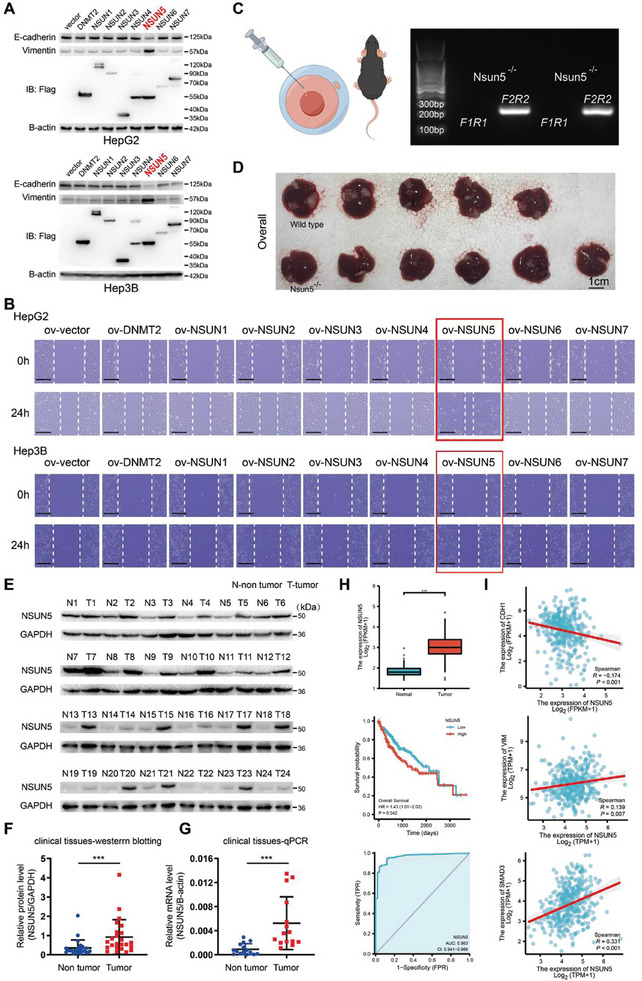
NSUN5 is highly expressed in hepatocellular carcinoma (HCC) tumor tissues. A) Western blotting was used to investigate the relationship between overexpression of several proteins potentially mediating RNA 5‐methylcytosinylation and epithelial–mesenchymal transition (EMT) in HepG2 and Hep3B cells. B) Results from wound healing assays indicated a significantly enhanced migration rate of HCC cells only upon overexpression of NSUN5. C) Homozygous Nsun5‐deficient mice were identified following genotyping. D) Modeling using CCL_4_ and DEN. After 16 weeks, analysis of liver tissues from wild‐type (n = 5) and Nsun5‐knockout (KO, n = 6) mice revealed a significant reduction in both the number and volume of tumor tissues in the KO group. E) Western blot analysis of NSUN5 levels in paired tumor and nontumor tissues, with GAPDH used as the loading control. F) Statistical analysis of western blot results from clinical samples demonstrated a significant increase in NSUN5 expression in HCC tumor tissues. G) qPCR assays of NSUN5 expression in paired tumors and corresponding paracancerous tissues showed significantly elevated NSUN5 mRNA levels in HCC tumor tissues, with β‐actin used as the control. H) Analysis of data from patients with HCC in TCGA database revealed the following results: NSUN5 was highly upregulated in tumor tissues compared to paracancerous tissues (upper). High NSUN5 expression was negatively correlated with patient prognosis (middle). The area under the receiver operating characteristic (ROC) curve (AUC > 0.9) indicated high predictive accuracy of NSUN5 for patient prognosis (lower). I) Analysis of TCGA database revealed that NSUN5 expression in HCC tumor tissues was inversely correlated with the expression of the epithelial marker E‐cadherin and positively correlated with that of the mesenchymal marker vimentin. Moreover, increased NSUN5 expression was significantly correlated with elevated SMAD3 expression in HCC. Results in (F) and (G) are shown as mean ± SD. P‐values are indicated by * < 0.05; ** < 0.01; *** < 0.001.

To assess whether proteins involved in 5‐methylcytosinylation of RNA mediate EMT in HCC, we transfected DNMT2 and NSUN1‐7 overexpression plasmids, along with their corresponding empty control plasmids, into HepG2 and Hep3B cells, respectively, to establish stable cell lines. Western blotting analysis of representative EMT markers (E‐cadherin and vimentin) indicated that significant EMT occurred in HepG2 and Hep3B cells only when NSUN5 was overexpressed (**Figure** **1**A). Furthermore, wound healing assays demonstrated enhanced migration ability of HCC cells exclusively upon NSUN5 overexpression (Figure 1B). Given that NSUN5 overexpression promotes cell motility in HCC, coupled with changes in EMT markers, we hypothesized that NSUN5 functions as a key promoter of EMT in HCC. To further investigate its role in HCC progression, we generated an Nsun5‐knockout (KO) mouse model (Figure S1A, Supporting Information; Figure 1C). Following induction with DEN and CCL_4_, we observed significantly reduced tumor size and number in the livers of Nsun5‐KO mice compared to wild‐type mice, confirming the involvement of NSUN5 in HCC malignancy (Figure 1D). To further elucidate its role in HCC, we quantified NSUN5 expression in tumor tissues relative to adjacent tissues using quantitative reverse transcriptase polymerase chain reaction (qRT‐PCR) and western blotting assays. Western blotting revealed higher NSUN5 expression in tumor tissues compared to corresponding nontumor tissues (Figure 1E,F). In another set of samples, qRT‐PCR demonstrated significantly higher NSUN5 expression in tumor tissues than in normal tissues (Figure 1G), indicating that high NSUN5 expression may hold clinical significance in HCC. Furthermore, analysis of HCC data from The Cancer Genome Atlas (TCGA) database revealed that NSUN5 is markedly upregulated in tumor tissues compared to adjacent tissues. High NSUN5 expression was negatively correlated with patient prognosis, as evidenced by a high area under the receiver operating characteristic curve (AUC = 0.963), indicating robust molecular prediction accuracy (Figure 1H). Analysis of the correlation between NSUN5 expression and EMT markers in HCC revealed that increased NSUN5 expression corresponded with decreased levels of E‐cadherin and continuous elevation of vimentin levels (Figure 1I). These findings confirm our center's data and underscore the pivotal role of NSUN5 in the onset and progression of HCC.

### 2.2 NSUN5 Actively Promotes EMT in HCC

**Figure 2:**
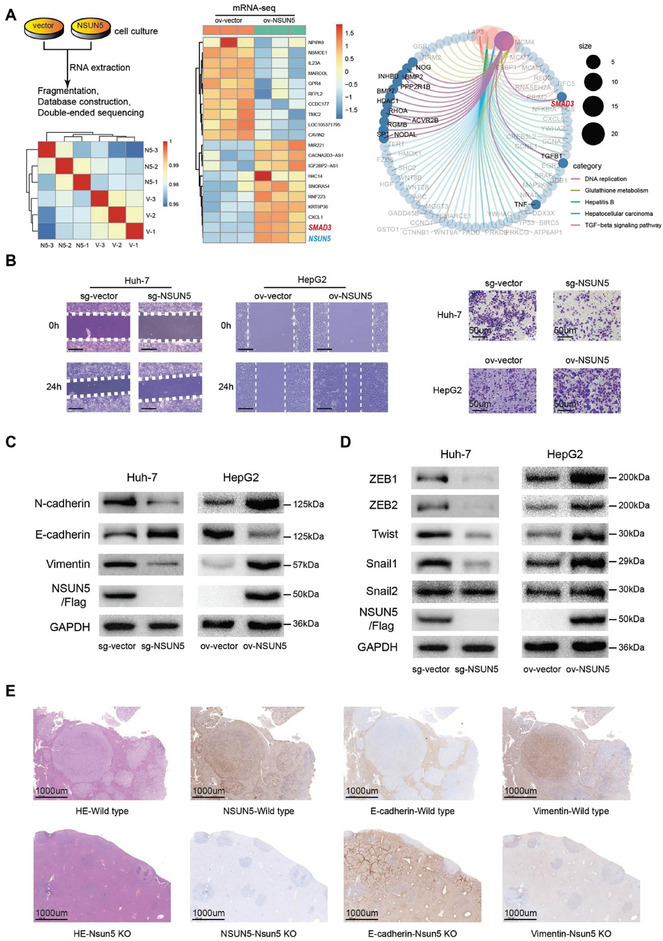
Upregulation of NSUN5‐induced EMT in HCC cells. A) Transcriptome analysis showed that Gene Ontology enrichment confirmed the involvement of NSUN5 in various biological behaviors, including mediating EMT. Kyoto Encyclopedia of Genes and Genomes signaling pathway and differential gene expression analyses suggested that NSUN5 could influence the SMAD3 signaling pathway by regulating SMAD3 expression. B) Results from wound healing and transwell assays demonstrated that higher NSUN5 expression enhanced the invasiveness and metastatic ability of HCC cells and vice versa. C) Western blot analysis of epithelial (E‐cadherin) and mesenchymal (vimentin and N‐cadherin) markers in Huh‐7‐sg‐vector/NSUN5 and HepG2‐ov‐vector/NSUN5 cells. D) Western blot analysis of several major EMT transcription factors (ZEB1/2, Snail1/2, and Twist) in Huh‐7‐sg‐vector/NSUN5 and HepG2‐ov‐vector/NSUN5 cells. E) Immunohistochemical (IHC) staining showed that Nsun5 knockout was accompanied by increased tumor epithelial marker (E‐cadherin) and decreased mesenchymal marker (vimentin) levels in spontaneous mouse HCC tissues.

To further analyze the role of NSUN5 in HCC, we used qRT‐PCR to detect NSUN5 expression levels across normal cells and various HCC cell lines. Consistent with previous findings, NSUN5 expression was generally elevated in HCC cell lines. Specifically, NSUN5 expression was highest in Huh‐7 cells and lowest in HepG2 cells (Figure S2A, Supporting Information). Consequently, we established stable cell lines using Huh‐7 and HepG2 cells (**Figure** **2**C). Transcriptome sequencing was performed to elucidate the role of NSUN5 in HCC (Figure 2A). The results from high‐throughput sequencing indicated that NSUN5 potentially mediates EMT in HCC (Figure 2A), corroborating the experimental findings shown in Figure 1. Given that EMT typically enhances cell invasion and migration, we performed wound healing (Figure 2B) and transwell assays (Figure 2C) on the established stable cell lines. The results revealed that HepG2 cells overexpressing NSUN5 (HepG2‐ov‐NSUN5) exhibited higher invasion and migration abilities compared to the corresponding control group, whereas Huh‐7 cells with NSUN5 KO (Huh‐7‐sg‐NSUN5) showed reduced invasive and migration abilities. To validate the involvement of NSUN5 in EMT, we further examined the expression of EMT markers using western blotting in KO and overexpression cell lines. NSUN5 overexpression decreased the expression of epithelial markers (E‐cadherin) and increased mesenchymal markers (vimentin and N‐cadherin) in HepG2 cells. Conversely, NSUN5 KO in Huh‐7 cells led to a significant increase in epithelial marker levels and a decrease in mesenchymal marker levels. These findings demonstrated that NSUN5 can induce EMT in HCC (Figure 2C). Further experiments also showed that NSUN5 KO or overexpression in HCC cell lines resulted in corresponding decreases or increases in the expression levels of transcription factors directly involved in mediating EMT (Figure 2D).

To further verify these findings, we generated primary mouse embryonic fibroblasts (MEFs) from Nsun5^−/−^ KO mice and wild‐type mice separately. As shown in Figure S2B,C (Supporting Information), wound healing (left) and transwell (middle) assays demonstrated significantly reduced invasion and migration abilities of MEFs following Nsun5 KO.

Prior research has demonstrated that the EMT state in HCC can impact the responsiveness to anti‐tumor drugs.^[^ ^29^ ^]^ Therefore, we utilized the stable transfected cell lines mentioned above to carry out cell viability experiments with sorafenib (10 um) for further investigation. The findings revealed that following NSUN5 knockout, the sensitivity of Huh‐7 cells to sorafenib increased. Conversely, in HepG2 cells which had undergone EMT through NSUN5 overexpression, resistance of HepG2‐ov‐NSUN5 to sorafenib was significantly augmented (Figure S2D, Supporting Information).

Subsequently, we employed immunohistochemical (IHC) staining to confirm whether Nsun5 KO significantly increased E‐cadherin expression in DEN/CCL_4_‐induced mouse HCC tissues, accompanied by a decrease in the expression of the mesenchymal marker vimentin (Figure 2E). These results, combined with the corresponding sequencing data and previous experimental findings, collectively support the conclusion that elevated NSUN5 expression can promote EMT in HCC, prompting further exploration into the underlying molecular mechanisms.

### 2.3 NSUN5 Promotes EMT in HCC by Increasing Intracellular SMAD3 Levels

**Figure 3:**
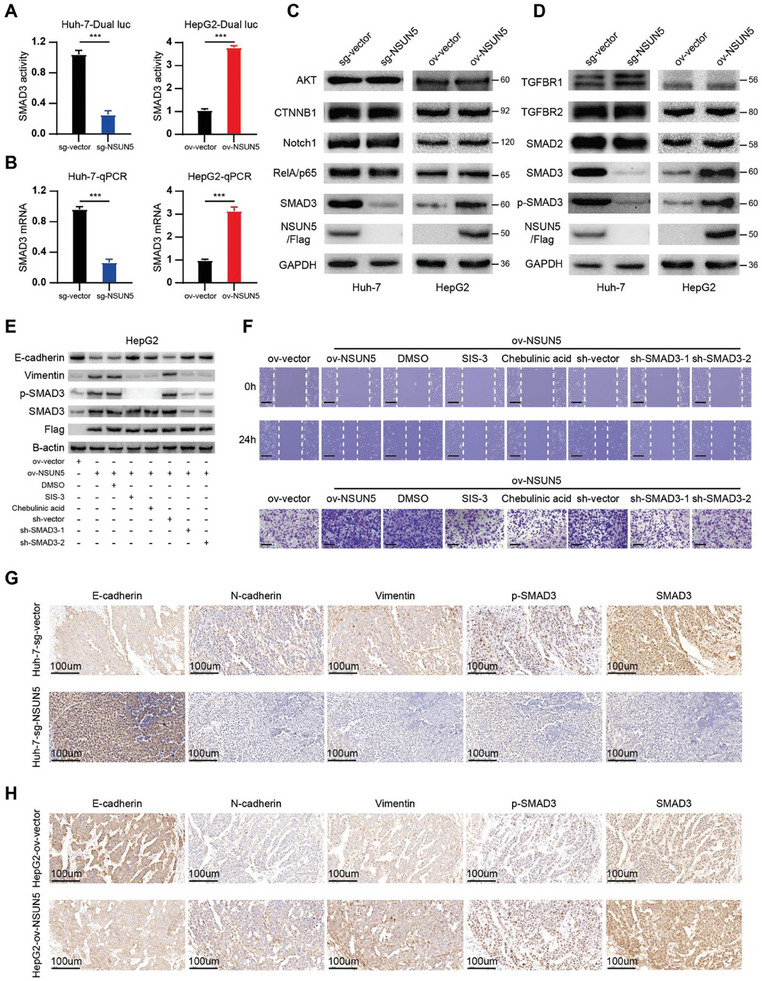
Upregulation of NSUN5 increases SMAD3 expression to induce EMT. A) Dual‐luciferase reporter gene assays revealed significant downregulation of the SMAD3 signaling pathway in Huh‐7 cells after NSUN5 knockout, whereas it was markedly upregulated after NSUN5 overexpression in HepG2 cells (n = 3, ***P < 0.001). B) qPCR analyses demonstrated a significant increase in SMAD3 mRNA content in HepG2 cells following NSUN5 overexpression, whereas the opposite trend was observed in Huh‐7 cells (n = 3, ***P < 0.001). C) Western blot analysis of key molecules involved in signaling pathways that induce major EMT transcription factors revealed that NSUN5 mainly affected the SMAD3 signaling pathway without affecting the expression of other key transcription factors. D) Western blot analysis of key molecules in the SMAD3 signaling pathway showed that NSUN5 predominantly affected SMAD3 expression and the levels of its active form, p‐SMAD3, without significantly altering the levels of other important molecules. E) Western blot analysis demonstrated that decreased epithelial marker (E‐cadherin) and increased mesenchymal marker (vimentin and N‐cadherin) levels could be rescued by SMAD3 knockdown or inhibition in HepG2‐ov‐NSUN5 cells. F) Wound healing and transwell assays confirmed that the enhanced invasive and migration ability of HepG2 cells due to NSUN5 overexpression was dependent on the activation of the SMAD3 signaling pathway. G) IHC staining of SMAD3 and EMT markers in subcutaneous tumors showed that NSUN5 knockout suppressed the activity of the SMAD3 signaling pathway, accompanied by significant progression of MET in the Huh‐7 cells group (n = 5 per group). H) IHC staining of SMAD3 and EMT markers in subcutaneous tumors demonstrated that NSUN5 overexpression upregulated the SMAD3 signaling pathway, accompanied by significant progression of EMT in HepG2 cells (n = 5 per group). Results in (A) and (B) are shown as mean ± SD. P‐values are indicated by * < 0.05; ** < 0.01; *** < 0.001.

Transcriptome sequencing results revealed a significant increase in SMAD3 mRNA levels upon NSUN5 overexpression in HepG2 cells, thereby activating the SMAD3 signaling pathway (Figure 2A). Considering the crucial role of this pathway in EMT, we further validated these findings using dual‐luciferase reporter gene and qPCR assays. NSUN5 KO inhibited SMAD3 signaling activity and reduced intracellular SMAD3 mRNA levels in Huh‐7 cells, whereas NSUN5 overexpression had the opposite effect in HepG2 cells (**Figure** **3**A,B). Additionally, western blotting revealed that among several key molecules involved in EMT, NSUN5 specifically modulates SMAD3 expression levels (Figure 3C), without affecting the abundance of other critical proteins involved in the SMAD3 signaling pathway (Figure 3D). To confirm that NSUN5 promotes EMT in HCC through SMAD3, we conducted rescue experiments using small‐hairpin RNA (shRNA) targeting SMAD3 and specific SMAD3 inhibitors. Specific shRNAs targeting SMAD3 were used to suppress the elevated SMAD3 expression induced by NSUN5 overexpression, or SIS‐3/chebulinic acid was administered to inhibit the phosphorylated active form of SMAD3 without reducing its total expression. These interventions restored the altered expression levels of epithelial and mesenchymal markers resulting from NSUN5 overexpression (Figure 3E). Wound healing and transwell assays showed that knocking down SMAD3 or inhibiting its phosphorylated active form attenuated the increased invasion and migration abilities observed upon NSUN5 overexpression (Figure 3F). Subsequently, tumor formation experiments in nude mice revealed that NSUN5 KO significantly reduced SMAD3 and p‐SMAD3 levels in tumors, accompanied by an increase in E‐cadherin (an epithelial marker) and a decrease in vimentin (a mesenchymal marker) (Figure 3G). Conversely, NSUN5 overexpression significantly enhanced the SMAD3 signaling pathway in tumors and advanced the EMT process, thereby confirming the results of the cellular experiments (Figure 3H). In conclusion, these findings indicate that increased NSUN5 expression promotes the initiation and progression of HCC by upregulating SMAD3 expression to drive EMT.

### 2.4 NSUN5 Binds to the DNA Region of SMAD3 and Enhances its Accessibility to Increase SMAD3 Expression

**Figure 4:**
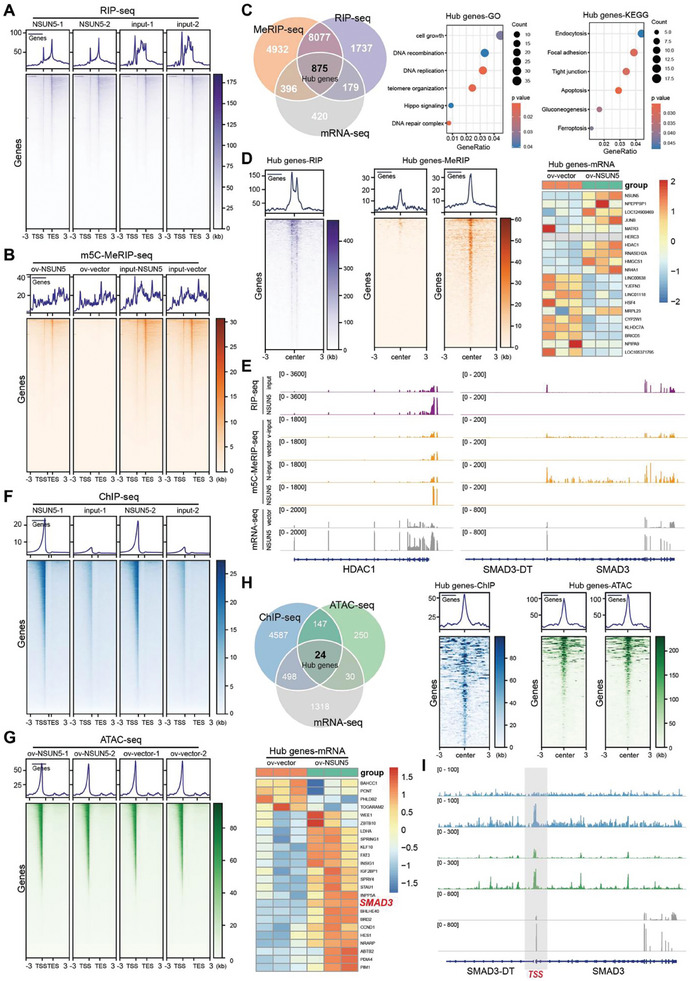
NSUN5 binds to the DNA promoter region of SMAD3 and enhances its accessibility to increase the expression of SMAD3. A) RNA immunoprecipitation (RIP) sequencing was applied to investigate the mRNA bound to NSUN5 in HepG2‐ov‐NSUN5 cells. B) Anti‐m5C methylated RNA immunoprecipitation (m5C meRIP) sequencing was used to detect the distribution and composition of m5C modifications of mRNA in HepG2‐ov‐NSUN5 and corresponding control cells before and after NSUN5 overexpression. C) Integration of mRNA‐seq, RIP‐seq, and meRIP‐seq data showed that NSUN5 exerts regulatory control over mRNA content in HCC cells by interacting with numerous mRNAs (n = 875) and influencing their m5C modification. D) NSUN5, an m5C writer, may not regulate all mRNAs. The results also revealed that the expression of mRNAs interacting with NSUN5 and their m5C abundance can be positively or negatively regulated. E) Results showed direct binding between NSUN5 and HDAC1 mRNA, significantly increasing the m5C modification of HDAC1 mRNA upon NSUN5 overexpression in HCC cells, followed by an increase in HDAC1 mRNA abundance. However, despite NSUN5 overexpression enhancing SMAD3 content in HCC cells, no direct interaction was observed between these two factors. F) ChIP‐seq was applied to investigate the genomic DNA distribution of NSUN5 in HepG2‐ov‐NSUN5 cells and analyze its localization pattern in HCC. G) ATAC‐seq was employed to examine alterations in chromatin accessibility in HCC cells following NSUN5 overexpression in HepG2‐ov‐NSUN5 and control cells. H) Integrated analysis using ChIP‐seq, ATAC‐seq, and mRNA‐seq revealed that NSUN5 exerts regulatory control over chromatin accessibility by directly binding to genomic DNA, thereby influencing the transcriptional regulation of specific genes, including SMAD3. I) IGV visualization results further validated the direct binding of NSUN5 to the transcription start site (TSS) of SMAD3 DNA, facilitating accessibility to the SMAD3 DNA region and promoting increased SMAD3 mRNA expression in HCC.

We employed various high‐throughput sequencing techniques to explore the underlying molecular mechanisms through which NSUN5 promotes EMT in HCC cells. Given that NSUN5 is an RNA‐binding protein implicated in the m5C modification of mRNA, we initially conducted RIP‐seq analysis in HepG2‐ov‐NSUN5 cells to identify potential mRNA targets of NSUN5 (**Figure** **4**A). Additionally, meRIP‐seq analysis was performed in HepG2‐ov‐NSUN5 cells to investigate changes in the distribution and abundance of m5C on mRNA before and after NSUN5 overexpression, with nontransfected HepG2 cells serving as controls (Figure 4B). These analyses revealed that NSUN5 interacts with numerous mRNAs in HCC cells, leading to alterations in m5C modifications across these transcripts (Figure 4A,B). To further investigate the potential of NSUN5 as an RNA‐binding protein in regulating SMAD3 mRNA, we integrated mRNA‐seq data with RIP‐seq and meRIP‐seq results. The findings revealed that NSUN5 regulates mRNA content in HCC cells by interacting with numerous mRNAs (n = 875) and influencing their m5C modification, thereby participating in various biological processes and mediating multiple signaling pathways in HCC (Figure 4C). Additionally, our results suggest that NSUN5's role as an m5C‐writer does not universally apply to all mRNAs (Figure 4C,D). Interestingly, the expression of mRNAs interacting with NSUN5 and their m5C modification levels can exhibit both positive and negative regulatory patterns (Figure 4D).

For instance, our findings revealed a direct binding between NSUN5 and HDAC1 mRNA, resulting in a significant increase in m5C modification of HDAC1 mRNA upon NSUN5 overexpression in HCC cells, followed by elevated HDAC1 mRNA expression (Figure 4E). In contrast, despite the ability of NSUN5 overexpression to enhance SMAD3 levels in HCC cells, no direct interaction between NSUN5 and SMAD3 was observed (Figure 4E), indicating that NSUN5 may influence SMAD3 expression in HCC through alternative mechanisms. After confirming the nuclear localization of NSUN5 using GeneCards (www.genecards.org), we performed ChIP‐seq analysis in HepG2‐ov‐NSUN5 cells, which revealed extensive binding of NSUN5 to genomic DNA (Figure 4F). Subsequently, ATAC‐seq was conducted in both HepG2‐ov‐NSUN5 and control group cells to investigate alterations in chromatin accessibility following NSUN5 overexpression (Figure 4G). Integrated ChIP‐seq, ATAC‐seq, and mRNA‐seq analysis demonstrated that NSUN5 regulates chromatin accessibility through direct genomic DNA binding, influencing transcriptional regulation of specific genes including SMAD3 (Figure 4H). Visualization results confirmed the direct binding of NSUN5 to the SMAD3 DNA region (transcription start site, TSS), thereby enhancing SMAD3 DNA accessibility and promoting increased SMAD3 mRNA expression in HCC (Figure 4I).

### 2.5 NSUN5 Recruits WDR5 to Increase SMAD3 Expression

**Figure 5:**
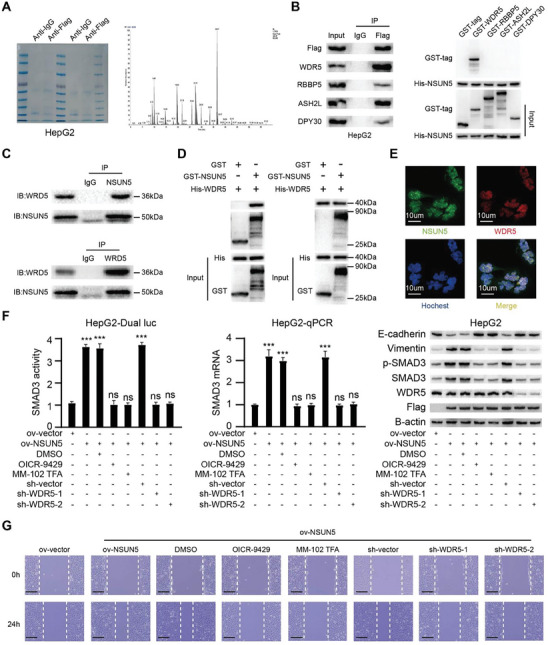
NSUN5 recruits WDR5 to increase SMAD3 expression. A) Cellular extracts expressing Flag‐NSUN5 were immunopurified using anti‐Flag, separated via SDS‐PAGE, and subjected to Coomassie brilliant blue staining to verify successful immunoprecipitation (IP) (left). Cellular extracts were then analyzed via LC–MS/MS to explore potential interacting partners of NSUN5 (right). B) Through co‐immunoprecipitation (Co‐IP) and pulldown assays, we speculated that NSUN5 directly interacts with WDR5 to facilitate recruitment of the lysine methyltransferase complex. C) Endogenous Co‐IP assays confirmed that NSUN5 interacts with WDR5 in HCC cells, consistent with previous mass spectrometry findings. D) GST‐pulldown and His‐pulldown assays demonstrated that NSUN5 can directly bind to WDR5 to form heterodimers. E) Endogenous immunofluorescence staining showed that NSUN5 was distributed in the nucleus and cytoplasm, significantly colocalizing with WDR5 in the nucleus. F) Rescue experiments revealed that downregulating WDR5 expression or inhibiting its function significantly attenuated the upregulation of the SMAD3 signaling pathway and elevated levels of SMAD3 mRNA induced by NSUN5 overexpression. Additionally, the aberrant EMT indices induced by NSUN5 overexpression returned to normal. G) Wound healing assays revealed that the enhanced migration ability of HCC cells due to NSUN5 overexpression was dependent on its interaction with WDR5. Results in (F) are shown as mean ± SD. P‐values are indicated by * < 0.05; ** < 0.01; *** < 0.001.

To further elucidate how NSUN5 increases SMAD3 expression, we conducted structural analysis of NSUN5 but did not identify any latent structural domains that could regulate chromatin conformation. Therefore, we employed co‐immunoprecipitation (Co‐IP) assays to identify the interacting partners of NSUN5. Clear bands were detected following Coomassie brilliant blue staining and subjected to LC–MS/MS, revealing WDR5 and RBBP5 as potential interactors of NSUN5 (**Figure** **5**A). Both WDR5 and RBBP5 are components of the lysine methyltransferase complex, which includes WDR5, RBBP5, ASH2L, and DYP‐30 as core components catalyzing H3K4 methylation and activating gene transcription for epigenetic regulation. Initially, Co‐IP and pulldown assays indicated interaction between NSUN5 and these proteins in HepG2 cells. Subsequent pulldown experiments showed that NSUN5 directly bound to WDR5 alone, indicating that WDR5 may serve as a direct interacting partner of NSUN5 (Figure 5B). Confirmation of endogenous NSUN5‐WDR5 interaction in Huh‐7 cells was achieved through Co‐IP assays (Figure 5C). Further pulldown assays and immunofluorescence experiments confirmed direct binding of NSUN5 to WDR5 in the nucleus, where they formed heterodimers (Figure 5D,E).

To confirm the role of WDR5 in mediating NSUN5‐dependent promotion of EMT in HCC, we utilized shRNA targeting WDR5 and specific WDR5 inhibitors in rescue experiments. Our results showed that using specific shRNAs to suppress WDR5 expression or introducing OICR‐9429/MM‐102 TFA to inhibit its function without reducing intracellular WDR5 content significantly attenuated the enhanced activity of the SMAD3 signaling pathway and elevated levels of SMAD3 mRNA induced by NSUN5 overexpression. Concurrently, the aberrant EMT markers triggered by NSUN5 overexpression returned to baseline levels (Figure 5F). Furthermore, wound healing assays confirmed that the increased migration ability of HCC cells due to NSUN5 overexpression was dependent on its interaction with WDR5 (Figure 5G). In conclusion, these findings demonstrate that NSUN5 promotes EMT in HCC by upregulating SMAD3 expression through its interaction with WDR5.

### 2.6 NSUN5 Recruits WDR5 and Activates the SMAD3 Signaling Pathway through its Interaction Structural Domain to Promote EMT in HCC

**Figure 6:**
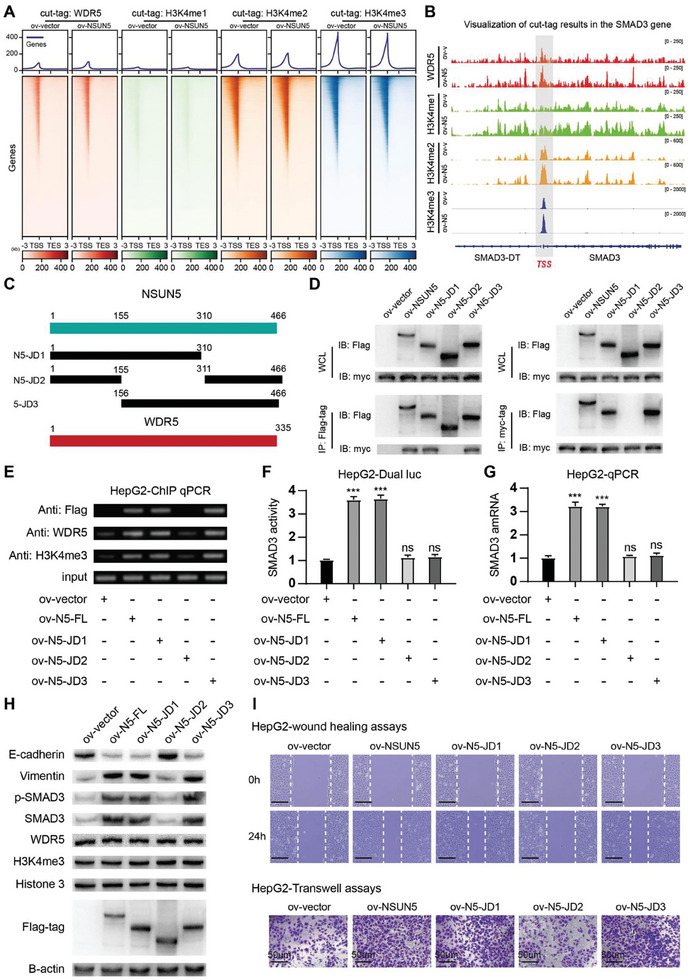
The 155–311 amino acid (aa) sequence of NSUN5 is indispensable for the function of the NSUN5‐WDR5 interaction. A) We employed a cut‐tag assay to assess alterations in WDR5 and H3K4me1/2/3 enrichment at the TSS of SMAD3 before and after NSUN5 overexpression in HCC cells. B) Visualization results demonstrated significant enhancement in the enrichment of WDR5 and H3K4me1/2/3 within the DNA promoter region of SMAD3 following NSUN5 overexpression in HCC cells. C) To explore the interaction domain between NSUN5 and WDR5, we constructed various truncated forms of NSUN5 using the pcDNA3.1‐Puro‐C‐3Flag plasmid. D) Using Co‐IP assays, we identified that the middle segment of NSUN5 (155–311 aa region, IS domain) is crucial for its interaction with WDR5 (right). E) ChIP‐qPCR analysis revealed that the binding of NSUN5 to the SMAD3 DNA region is dependent on its IS domain. The enrichment of WDR5 and H3K4me3 in the SMAD3 promoter region was not significant in the absence of this domain. F) Dual‐luciferase reporter gene assay showed that overexpression of the NSUN5‐JD2 truncated form did not upregulate the SMAD3 signaling pathway. G) qPCR assays demonstrated that the IS domain is a key domain on NSUN5 that promotes the increase in SMAD3 mRNA content in HCC cells. H) Western blot analysis demonstrated that when only the JD2 truncation (deletion of the 155–311 amino acid sequence) was overexpressed, the EMT indicators of HCC cells did not change significantly, revealing that the middle segment of NSUN5 is crucial for promoting the EMT process in HCC cells. I) Wound healing and transwell assays demonstrated that the IS domain of NSUN5 is required to enhance the invasion and migration abilities of HCC cells. Results in (F) are shown as mean ± SD. P‐values are indicated by * < 0.05; ** < 0.01; *** < 0.001.

A previous study highlighted WDR5 as a crucial regulator of histone H3K4 methylation in eukaryotic cells.^[^ ^30^ ^]^ Expanding on our earlier findings regarding the formation of NSUN5‐WDR5 heterodimers and their collaborative regulation of SMAD3 expression in HCC cells, we investigated whether elevated NSUN5 expression in HCC affects H3K4 methylation via its interaction with WDR5, thereby exerting a corresponding promotional effect. To explore this, we performed cut‐tag assays to assess WDR5 and H3K4me1/2/3 enrichment in HepG2‐ov‐NSUN5 cells and their respective controls (**Figure** **6**A). Subsequent visualization results revealed significant enrichment of these markers within the SMAD3 TSS region in HepG2‐ov‐NSUN5 cells following NSUN5 overexpression compared to the control group (Figure 6B).

To identify the specific WDR5‐interacting region within NSUN5, we generated a series of expression plasmids containing truncated NSUN5 variants tagged with FLAG (5‐JD1, 5‐JD2, 5‐JD3) (Figure 6C). Each plasmid was co‐transfected into HepG2 cells along with a WDR5‐Myc‐tag expression plasmid. Co‐IP results revealed that the amino acid residues 155–311, constituting the interaction structural (IS) domain of NSUN5, were essential for its interaction with WDR5 (Figure 6D), as confirmed using pulldown assays (Figure S6A, Supporting Information). Subsequent ChIP‐qPCR analyses demonstrated that NSUN5 specifically relies on its IS domain to bind to the SMAD3 DNA region. Notably, overexpression of the truncated NSUN5‐JD2 form lacking this domain failed to recruit WDR5 and H3K4me3, leading to compromised enrichment at the SMAD3 DNA locus (Figure 6E). These findings underscore the essential role of the IS domain in NSUN5's interaction with SMAD3 DNA and its ability to recruit WDR5 through heterotrimer formation. Further investigations were conducted to validate the role of the IS domain in the NSUN5‐mediated promotion of EMT in HCC. First, results from dual‐luciferase reporter gene and qPCR assays indicated that overexpression of the NSUN5‐JD2 truncated form did not elevate SMAD3 signaling pathway activity or intracellular SMAD3 mRNA content (Figure 6F,G). Second, western blotting revealed no significant changes in EMT markers when NSUN5 lacked the IS domain in HCC cells (Figure 6H). Finally, wound healing and transwell assays demonstrated that NSUN5 relied on its IS domain to enhance the invasion and migration abilities of HCC cells (Figure 6I). Therefore, we conclude that the IS domain is vital for facilitating NSUN5‐mediated recruitment of WDR5 to the SMAD3 DNA region. This interaction potentially enhances the accessibility of this region, thereby activating the SMAD3 signaling pathway to promote EMT in HCC.

### 2.7 Overexpression of NSUN5 In Vivo Promotes HCC Progression

**Figure 7:**
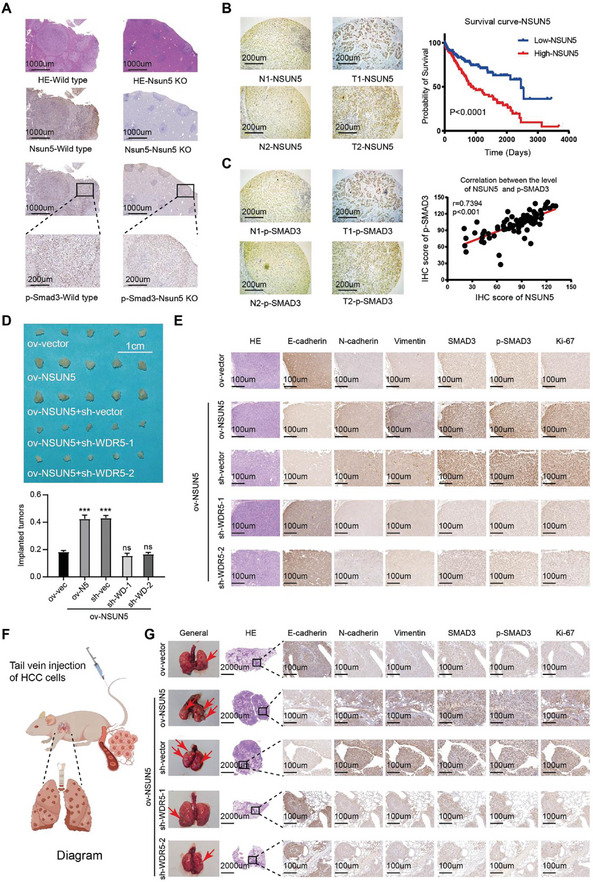
Overexpression of NSUN5 in vivo promotes the course of HCC. A) Hematoxylin and eosin (H&E) staining and IHC staining were used in spontaneous mouse HCC tissues. Experimental results revealed significant downregulation of the SMAD3 signaling pathway (indicated by p‐SMAD3) upon NSUN5 knockout. B) IHC staining results from the tissue microarray confirmed the increased expression of NSUN5 in HCC tumor tissues compared with that in adjacent tissues, and this high expression was often accompanied by poor prognosis in these patients. C) Using the same set of tissue microarrays, we found that p‐SMAD3 expression was higher in the NSUN5‐high group, validating our aforementioned findings and confirming a clinically significant correlation between NSUN5 and SMAD3 activation. D) Subcutaneous tumorigenic experiments in nude mice showed that NSUN5 overexpression resulted in increased HCC proliferation, which was significantly reduced by decreasing WDR5 content in HepG2‐ov‐NSUN5 cells (n = 5 per group). E) Corresponding IHC staining revealed increased SMAD3 expression in NSUN5‐overexpressing tumors, leading to enhanced SMAD3 signaling pathway (indicated by p‐SMAD3). This promoted EMT in HCC, which could be reversed upon knocking down WDR5. F) Tail vein metastasis mouse model (n = 5 per group). G) Lung metastases in mice injected with HepG2‐ov‐NSUN5 cells were more numerous and larger than those in the corresponding negative control group in the gross specimens, which was further confirmed through H&E and IHC staining. Results in (D) are shown as mean ± SD. P‐values are indicated by * < 0.05; ** < 0.01; *** < 0.001.

As described above, NSUN5 accelerates HCC malignancy by activating the SMAD3 signaling pathway at the cellular level. To further substantiate these findings in vivo, we performed IHC staining on liver cancer specimens induced in wild‐type and Nsun5‐KO mice, which revealed a significant reduction in Smad3 content in Nsun5^−/−^ mice, consistent with our experimental observations (**Figure** **7**A). Additionally, to explore the clinical relevance of the NSUN5/WDR5‐SMAD3‐EMT axis, we conducted further verification using tissue microarrays. IHC staining confirmed elevated NSUN5 expression in HCC tumor tissues compared to adjacent tissues, often associated with poor patient prognosis (Figure 7B). Additionally, analysis of the same tissue microarrays showed increased expression of p‐SMAD3 in the NSUN5‐high group, correlating with NSUN5 expression levels and confirming the clinical significance of NSUN5 in activating SMAD3 (Figure 7C). Furthermore, we investigated the role of NSUN5 in the malignant progression of HCC in vivo. First, using subcutaneous tumor formation in mice, we observed that NSUN5 overexpression increased HCC proliferation, an effect significantly attenuated by reducing WDR5 levels in HepG2‐ov‐NSUN5 cells (Figure 7D). Corresponding IHC staining showed that NSUN5‐overexpressing tumors exhibited elevated SMAD3 protein expression, leading to enhanced SMAD3 signaling pathway activity (p‐SMAD3), thereby promoting EMT in HCC. These effects were reversed upon WDR5 knockdown, highlighting the dependency of NSUN5‐induced EMT on WDR5 expression (Figure 7E). Second, in a tail vein metastasis mouse model (Figure 7F), mice injected with HepG2‐ov‐NSUN5 cells developed more and larger lung metastases compared to the negative control group, as confirmed by gross specimen examination and hematoxylin–eosin (H&E) staining. However, knocking down WDR5 in NSUN5‐overexpressing HCC cells significantly reduced the number and size of metastatic lesions in the lungs, accompanied by decreased metastatic tumor volume in the lungs, as validated via IHC staining (Figure 7G). Overall, these in vivo and clinical experiments provide compelling evidence supporting the role of the NSUN5/WDR5‐SMAD3‐EMT axis in promoting the malignant progression of HCC, underscoring its significant clinical implications.

### 2.8 NSUN5 can be Used as a Potential Therapeutic Target for HCC

**Figure 8:**
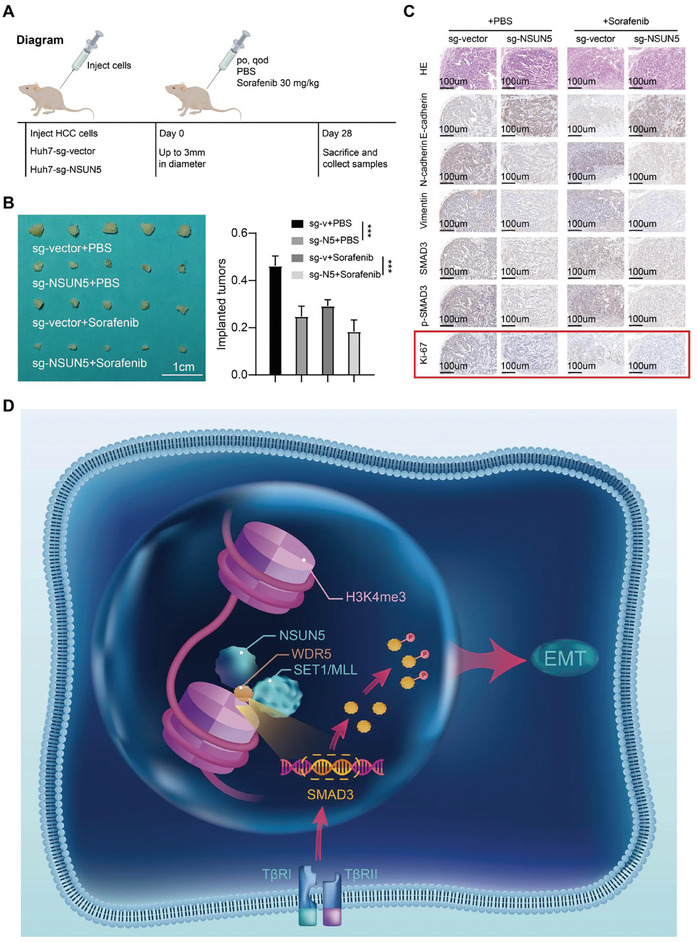
NSUN5 facilitates HCC progression by increasing SMAD3 expression. Schematic diagram demonstrating further experiments to explore the potential applicability of NSUN5 knockout for treating HCC. A) Compared with the corresponding control cells, Huh‐7‐sg‐NSUN5 cells showed higher drug sensitivity when Sorafenib (30 mg kg^−1^) was added, as confirmed by the statistical results (n = 5 per group). B) Corresponding IHC staining of EMT markers (E‐cadherin and vimentin) and related proliferation indexes (Ki‐67) in different groups. C) A diagram summarizing the findings: NSUN5, overexpressed in HCC, uses its IS domain to bind to SMAD3 DNA and recruit WDR5, thereby synergistically promoting the enrichment of H3K4me3 at the SMAD3 promoter. This enhances the accessibility of this DNA region and increases the expression of SMAD3 in HCC cells, facilitating EMT mediated by the SMAD3 signaling pathway to accelerate the progression of HCC. Results in (B) are shown as mean ± SD. P‐values are indicated by * < 0.05; ** < 0.01; *** < 0.001.

As stated above, elevated NSUN5 expression in HCC promotes its malignant progression by enhancing EMT. Several studies have highlighted the role of EMT in facilitating drug resistance in tumors.^[^ ^31^ ^]^ Based on this association, we investigated the potential therapeutic implications of targeting NSUN5 in HCC treatment. We administered sorafenib (30 mg kg^−1^) intragastrically to the Huh‐7‐sg‐vector/NSUN5 group (**Figure** **8**A). Our in vivo results demonstrated that combining sorafenib with NSUN5 KO significantly enhanced the therapeutic efficacy of sorafenib (Figure 8B), as confirmed via subsequent IHC staining (Figure 8C).

In conclusion, our findings establish NSUN5 as a promising therapeutic target for HCC treatment. We demonstrate that NSUN5, highly expressed in HCC, facilitates the accumulation of H3K4me3 at the SMAD3 promoter region by interacting with WDR5. This interaction enhances accessibility to the DNA, leading to increased SMAD3 expression and subsequent induction of EMT, thereby promoting distant metastasis in HCC (Figure 8D). These insights deepen our understanding of the molecular pathology underlying HCC and provide a foundational basis for identifying potential therapeutic targets in its treatment.

## 3 Discussion

Distant metastasis eliminates the possibility of radical surgery for patients with HCC, leading to a poor prognosis and often fatal outcomes.^[^ ^32^ ^]^ However, the drugs available for effectively treating patients with HCC are limited,^[^ ^33^ ^]^ prompting us to investigate factors contributing to the occurrence and progression of HCC, especially those that promote metastasis.^[^ ^34^ ^]^ In this study, we identified NSUN5 as a promoter of HCC metastasis and explored its underlying mechanism.

EMT refers to the transformation of cell phenotype from epithelial to mesenchymal under the influence of specific factors. This process often leads to increased cell invasion and enhanced metastatic potential,^[^ ^35^ ^]^ facilitating distant metastasis of tumors.^[^ ^36^ ^]^ Several studies have highlighted a strong association between EMT and drug resistance. Analysis of tissue samples from a patient with lung cancer revealed acquired resistance to erlotinib, where aside from T790 M mutation and MET amplification, no other resistance mechanisms were identified. Similarly, in studies on gefitinib‐resistant HCC827 cells, phenotypic and molecular changes consistent with EMT were observed. Recent research also demonstrated that gefitinib‐resistant PC9 and HCC827 cells exhibited a mesenchymal phenotype characterized by decreased expression of E‐cadherin and increased expression of mesenchymal markers, such as N‐cadherin, confirming the occurrence of EMT. Additionally, cells resistant to gefitinib or osimertinib (AZD9291) displayed significant EMT characteristics. In the present study, using multiple cellular and animal models, we found that knocking out NSUN5 in HCC substantially reduced tumor proliferation and suppressed metastatic potential. Moreover, it sensitized HCC to sorafenib, suggesting that NSUN5 accelerates HCC malignancy by promoting EMT. The pivotal role of SMAD3 in EMT is well‐established.^[^ ^37^ ^]^ SMAD3 is phosphorylated (p‐SMAD3) in response to upstream signals, which in turn promotes the expression of downstream EMT‐related transcription factors.^[^ ^38^ ^]^ Therefore, reducing intracellular SMAD3 expression or its active form p‐SMAD3 can potentially reverse EMT in tumor cells. Our results demonstrated that NSUN5 KO significantly decreased intracellular SMAD3 and p‐SMAD3 levels, reversing EMT in tumor cells and significantly reducing HCC invasion and metastasis both in vitro and in vivo. This underscores NSUN5 as a promising therapeutic target for HCC.

WDR5, a crucial regulatory protein involved in mediating the tri‐methylation of histone H3 at lysine 4 (H3K4me3),^[^ ^39^ ^]^ regulates the enzymatic activity of MLL proteins through interactions with other components of the complex (RBBP5, ASH2L, and DPY30). This process facilitates the transcriptional activation of various proteins.^[^ ^40^ ^]^ Previous studies have categorized WDR5 as a member of the WD40 repeat domain protein family,^[^ ^41^ ^]^ characterized by its seven trans‐β‐propeller domains. Each propeller domain consists of four trans‐parallel β‐folds,^[^ ^42^ ^]^ beginning with a conserved serine‐histidine and ending with tryptophan‐aspartate (WD), with a molecular weight of ≈34 kD. These domains encircle each other to form a central cavity traversing the protein from top to bottom.^[^ ^43^ ^]^ Despite recent advancements in WDR5 inhibitors, several challenges remain.^[^ ^44^ ^]^ Existing molecules tend to have relatively uniform structure types, whether peptide or nonpeptide small molecule inhibitors, often containing strong basic groups such as piperazine, guanidine, or nitrogenous aromatic five‐membered heterocycles.^[^ ^45^ ^]^ These structural characteristics can lead to limitations in drug properties, such as in vivo metabolic stability, membrane permeability, and high polarity, particularly in multisubstituted phenylpiperazines.^[^ ^46^ ^]^ Furthermore, while certain WDR5 inhibitors show promising in vitro activities, their efficacy in vivo requires further validation.^[^ ^47^ ^]^ Therefore, investigating endogenous proteins that interact with WDR5 to regulate its function remains pivotal. In this study, we discovered that NSUN5 modulates SMAD3 expression by interacting with WDR5 through its IS domain, thereby regulating the enrichment of tri‐methyl‐histone H3 (Lys4) at the SMAD3 promoter region. Consequently, we speculated whether NSUN5 also affects the transcription of other genes by binding to WDR5 and whether NSUN5 contributes to the progression of additional malignant phenotypes in HCC via interactions with other proteins.

Recent studies have demonstrated that EMT is not simply a binary transition but rather a complex process that includes intermediate or hybrid partial EMT (pEMT) states, where cells exhibit both epithelial and mesenchymal characteristics simultaneously.^[^ ^48^ ^]^ EMT plays a crucial role in cancer progression, involving multiple intermediate states in epithelial cells^[^ ^49^ ^]^; however, the molecular characteristics remain unclear. These states involve the co‐expression of epithelial and mesenchymal markers, rather than solely mesenchymal markers, which correlates with poor prognosis in clinical settings.^[^ ^50^ ^]^ Cells can transition through one or more hybrid pEMT states, which often exhibit higher malignancy compared to fully epithelial or fully mesenchymal transitions.^[^ ^51^ ^]^ Moreover, the EMT state of cells is closely associated with tumor metastasis and resistance to drugs. Therefore, accurately determining the proportion of cancer cells in various EMT states is crucial for predicting patient prognosis.^[^ ^52^ ^]^ Our analysis revealed that partial EMT phenotypes may create a more favorable environment for tumor invasion and metastasis. NSUN5 may contribute to transitioning cells into these intermediate states, given that current experimental methods typically focus on total protein expression levels in cells and tissues to assess their status, whereas pEMT phenotypes involve changes occurring within subpopulations of individual cells. In our study, we observed dynamic chromatin alterations in the tumor microenvironment related to the activation levels of the SMAD3 signaling pathway or its DNA‐binding ability, regulated by the NSUN5–SMAD3 axis. Consequently, individual cells can display distinct intermediate states (pEMT) that facilitate HCC invasion and metastasis.

In conclusion, we found that NSUN5 was highly expressed in HCC tissues, as confirmed by multiple clinical sample analyses and data from the TCGA database. This elevated expression of NSUN5 correlates negatively with patient prognosis. Through a series of cellular and molecular biology experiments, complemented by results from multiple high‐throughput sequencing analyses, we demonstrated that high NSUN5 expression promotes the accumulation of H3K4me3 at the promoter region of SMAD3 by interacting with WDR5. This molecular mechanism promotes the EMT process by upregulating SMAD3 expression, thereby facilitating the initiation and metastasis of HCC. Animal experiments further supported these findings, showing that NSUN5 KO significantly reduced the invasion and metastasis abilities of HCC cells. These results underscore NSUN5 as a potential therapeutic target for HCC, offering a novel theoretical basis for the development of treatments aimed at improving patient outcomes.

## 4 Experimental Section

### Cell Culture

Human hepatocellular carcinoma (HCC) cell lines (Huh‐7, Hep‐3B, PLC/PRF/5, and HepG2) and normal liver cell lines (Lo2) were purchased from Shanghai Fuheng Technology Co., Ltd. They were cultured and passaged according to standard protocols in a humidified atmosphere containing 5% CO_2_ at 37 °C supplied with DMEM (Hyclone) or RPMI medium (Hyclone) containing 10% FBS (Gibco, Invitrogen). All cells underwent short tandem repeat (STR) identification and were regularly tested for the presence of Mycoplasma. Cell transfection was performed using Lipo8000 transfection reagent (C0533, Beyotime, Shanghai, China) following the manufacturer's instructions, and transfected cells were selected and maintained in the presence of hygromycin (200 µg mL^−1^), puromycin (1µg mL^−1^), G418 (1 mg mL^−1^), or blasticidin (10 µg mL^−1^).

### Western Blotting Assays

All cells and fresh tissues were lysed in radioimmunoprecipitation (RIPA) buffer (Beyotime, Shanghai, China). The lysed cells and tissues were analyzed quantitatively using bicinchoninic acid (BCA) (Biosharp, Shanghai, China). After denaturation reduction (WB2001, NCM), 8–15% SDS‐PAGE (sodium dodecyl sulfate‐polyacrylamide gel electrophoresis) separated an equal amount of protein from each sample. Each PAGE was transferred to PVDF (polyvinylidene fluoride) membrane. The membranes were incubated with different primary antibodies and corresponding secondary antibodies (Beyotime, Shanghai, China), diluted by Universal antibody diluent (WB500D, New Cell & Molecular Biotech): Anti‐NSUN5 (sc‐376147), Anti‐E‐cadherin (sc‐8426), Anti‐Vimentin (sc‐6260), Anti‐N‐cadherin (sc‐8424), Anti‐ZEB1 (sc‐515797), Anti‐ZEB2 (sc‐271984), Anti‐Twist (sc‐81417), Anti‐Snail1 (sc‐271977), Anti‐Snail2 (sc‐166476), Anti‐TGFBR1 (sc‐518018), Anti‐TGFBR2 (sc‐17792), Anti‐SMAD2 (sc‐101153), Anti‐SMAD3 (sc‐101154), Anti‐p‐SMAD3 (sc‐517575), Anti‐RelA (sc‐8008), Anti‐CTNNB1 (sc‐7963), Anti‐WDR5 (sc‐393080) were from Santa Cruz, Anti‐Notch1 (#3609) and anti‐AKT (#4691) were obtained from Cell Signaling Technology; Anti‐NSUN5 (ab121633) was obtained from Abcam; Anti‐GAPDH (60004‐1‐Ig) was purchased from Proteintech.

### RNA Extraction, Real‐Time PCR, and RNA‐Seq Analysis

Total RNA from hepatocellular carcinoma (HCC) cells and fresh tissues were extracted with RNA‐easy Isolation Reagent (Vazyme, Nanjing, China). After determining the quality and concentration of the total RNA by Nanodrop 2000 spectrophotometer (Thermo Fisher Scientific, USA). Hiscript III‐RT SuperMix for qPCR (+gDNA wiper) (Vazyme, Nanjing, China) was used for reverse transcription and AceQ. Universal SYBR qPCR Master Mix (Vazyme, Nanjing, China) was used for quantitative real‐time PCR on an ABI 7500 Real‐time PCR System (Applied Biosystems. Foster City, CA). mRNA of β‐actin served as the housekeeping gene. The primers used are listed as follows:

PCR‐NSUN5‐F: CGCTACCATGAGGTCCACTAC

PCR‐NSUN5‐R: GCATCTCGCACCACGTCTT

PCR‐SMAD3‐F: GCGTGCGGCTCTACTACATC

PCR‐SMAD3‐R: GCACATTCGGGTCAACTGGTA

PCR‐β‐actin‐F: TCCCTGGAGAAGAGCTACG

PCR‐β‐actin‐R: GTAGTTTCGTGGATGCCACA

### Plasmid Construction and Transfection

As described in the previous paper.^[^ ^53^ ^]^ Complementary DNA (cDNA) of NSUN5 was synthesized (M0492, NEB) and cloned into pcDNA3.1‐Puro‐C‐3Flag.

OV‐NSUN5‐F: ATGGGGCTGTATGCTGCAGC

OV‐NSUN5‐R: TGTGCAAGGCGGTGTGCA

OV‐5‐JD1‐F: ATGGGGCTGTATGCTGCAGC

OV‐5‐JD1‐R: TGGATCCTTCCTGCAGTGGC

OV‐5‐JD2‐F: GTAGTTGATCGGGTATGCCGAGCAGACAGCTGG

OV‐5‐JD2‐R: GCATACCCGATCAACTACATCATCGGAGCAGGT

OV‐5‐JD3‐F: ATGTTATTTCAAGAGACAAGGTTTCTCCTA

OV‐5‐JD3‐R: TGTGCAAGGCGGTGTGCA

Complementary DNA of WDR5 was produced (M0492, NEB) and cloned into pcDNA3.1‐G418‐C‐Myc.

OV‐WDR5‐F: ATGGCGACGGAGGAGAAGA

OV‐WDR5‐R: GCAGTCACTCTTCCACAGTTTAATTG

Knocking out of endogenous proteins was performed by LentiCRISPRv2 vectors.

sg‐NSUN5‐A: TTTTGACCCCGGAAGTCCGGG

sg‐NSUN5‐B: TCCGGGCTGACTCCTTCCTGG

Knockdown of endogenous proteins was accomplished using two short hairpin RNA (shRNA) oligonucleotides cloned into pLKO.1‐TRC‐mCherry‐BSD vectors:

sh‐WDR5‐1: CAGGGCCAGAATTCACATTAT

sh‐WDR5‐2: CCAGATGCCAATCACAGACAT

After sequencing to verify that the sequence was correct, Lipo8000 transfection reagent was used to transfer different plasmids into various cells and added corresponding antibiotics to screen and obtain stable cell lines.

### Transwell Assays and Wound Healing Assays

As described in the previous paper.^[^ ^54^ ^]^ The invasive ability of cells was evaluated using a transwell assay. Briefly, cells were seeded into the upper chamber with media containing 0.1% FBS for transwell assay, while 10% FBS was placed in the lower chamber (3422, Corning). Consequently, invasive cells were stained and photographed under a microscope (Olympus).

A linear wound is formed by scraping the plate with a yellow sterile pipette tip for cell migration assay, after incubating in 1% FBS cell culture medium, photograph and measure the width of the wound gap at different points in time.

### Immunofluorescence (IF) and Immunohistochemistry (IHC)

Cells were fixed for 30 min in 4% methanol‐free formaldehyde and permeabilized for 30 min in 1% Triton X‐100 at 4 °C. After three rinses with phosphate‐buffered saline (PBS), cells were blocked for 30 min with 5% BSA and incubated with an anti‐NSUN5 (1:200) and anti‐WDR5 (1:200) antibody in 5% BSA overnight at 4 °C, after which they were rinsed three times with PBS and incubated with the secondary antibodies (1:500) at room temperature for 1 h. Then, cells were washed with PBS three times, and the nucleus was stained with Hoechst and fixed it with fluorescent mounting fluid. Finally, images were visualized with Leica confocal microscope and analyzed.

IHC staining was performed: Formalin‐fixed, paraffin‐embedded tissue slides were dewaxed with xylene and rehydrated by a graded series of alcohols, followed by antigen retrieval and block with 5% BSA for 60 min. Incubation was carried out at 4 °C for overnight in a humidified chamber with the primary antibody diluted in 5% (v/v) normal goat serum; primary antibodies included: Anti‐E‐cadherin (1:100, sc‐8426, Santa cruz), Anti‐Vimentin (1:100, sc‐6260, Santa cruz), Anti‐N‐cadherin (1:100, c‐8424, Santa cruz), Anti‐SMAD3 (1:100, sc‐101154, Santa cruz), Anti‐p‐SMAD3 (1:100, sc‐517575, Santa cruz) and Anti‐Ki‐67 (1:2000, 28074‐1‐AP,Proteintech). (HRP‐labeled Goat Anti‐Mouse IgG(H+L) or HRP‐labeled Goat Anti‐Rabbit IgG(H+L) (Beyotime, Shanghai, China) were used per the manufacturer's recommendations. The antibody‐antigen complex was visualized using 3,3‐ Diaminobenzidine (DAB) staining.

Furthermore, Aipathwell software was used for analysis, an artificial intelligence‐based digital pathology image analysis software developed by Servicebio. It employs AI deep learning principles and is a data‐driven automated image analysis software. The specific process entails the following steps: 1) Tracking: Automatically identifies and delineates the test area within the tissue, which can also be manually specified based on specific requirements. 2) Color Selection: Automatically performs positive assessment using HSI, with manual adjustments available to accommodate specific circumstances. 3) Calculation: Automatically locates cell nuclei and expands cytoplasmic regions as per user preferences. It calculates the number and area of weak, moderate, and strong positive cells; aggregates integrated optical density (IOD) of the tissue; and computes other parameters such as tissue area. 4) Analysis: Calculates high‐magnification test areas. Upon completion, the software automatically derives results for each item based on original raw data and algorithm formulas, generating a comprehensive report. The formula for calculation is as follows: H‐SCORE = ∑(pi×i) = (percentage of weak intensity ×1) + (percentage of moderate intensity ×2)+(percentage of strong intensity ×3). Based on the statistical results obtained from NSUN5 staining, the 180 patients were divided into high and low expression groups (each group consisted of 90 patients).

### Co‐Immunoprecipitation (Co‐IP) and Mass Spectrometry (MS) Analysis

As described in the previous paper.^[^ ^55^ ^]^ For immunoprecipitation, cells were washed with PBS three times, whole‐cell lyses prepared in IP buffer (P0013, Beyotime, Shanghai, China), and mixed with agarose beads (sc‐2003, Santa Cruz). These cells were rotated for two hours at 4 °C, followed by centrifugation at 12 000 rpm for 5 min. The supernatant was removed and used for immunoprecipitation. Concurrently, agarose beads were coated with the corresponding antibody for 2 h at 4 °C, followed by extensive washing with IP buffer three times. Then, the precleared lysate was mixed with the antibody‐coated beads and incubated with rotation overnight (16 h), centrifugated at 12 000 rpm for 15 min. The precipitates were washed by pre‐cold IP buffer. Finally, all the supernatants were discarded and boiled the precipitates. Immunocomplexes were analyzed by SDS‐PAGE and immunoblotting with the corresponding antibody or analyzed by mass spectrometry (MS) (Shanghai Bioprofile Technology Company Ltd.), and the proteins with potential interactions with NSUN5 as determined by LC‐MS/MS are presented in the Supplementary Materials in the form of a list.

### Transcriptome Sequencing (mRNA‐Seq) and MeRIP‐Seq

Transcriptome sequencing (mRNA‐seq) was performed by Shanghai Majorbio Bio‐pharm Biotechnology Co., Ltd. (Shanghai, China), and MeRIP‐seq was commissioned to Suqian Jianweipu Technology Service Co., Ltd.

### RNA Immunoprecipitation (RIP) and RIP‐Seq

As described in the previous paper.^[^ ^56^ ^]^ Briefly, after formaldehyde crosslinking, 25 million cells were harvested per sample and washed with phosphate‐buffered saline (PBS). Cells were lysed with 1 mL RIPA buffer (P0013C; Beyotime) and 2000 U mL^−1^ RNase inhibitor (R0102; Beyotime). To fragment the RNA, the lysate was sonicated for 30 cycles of 30 s ON and 30 s OFF.

Input samples were prepared by saving 5% of the lysate and adding 1 mL of Freezol reagent (R711, Vazyme, Nanjing, China). The residual lysate sample was then centrifuged at 12 000 rpm for 15 min and subjected to immunoprecipitation using 50 µL A/G agarose (sc‐2003, Santa Cruz) coated with normal mouse IgG (A7028, Beyotime, Shanghai, China) or anti‐Flag (F1804, Sigma), which were immunoprecipitated with rotation at 4 °C overnight. Thereafter, beads were washed three times with cold RIPA buffer, and immunoprecipitated beads were digested with proteinase K in RIPA buffer supplemented with 1% SDS and 1.2 mg mL^−1^ proteinase K (ST532, Beyotime, Shanghai, China), and incubated for 1 h at 55 °C with shaking at 1200 rpm. Approximately 1 mL of Freezol reagent was added. Finally, RNA was extracted from both the input and immunoprecipitated RNA, including DNase I digestion, and subsequently used for qPCR and Next‐Generation Sequencing, the RIP‐seq databasesm were built by VAHTS Universal V10 RNA‐seq Library Prep Kit for Illumina (NR606, Vazyme Biotech Co.,Ltd), which were sequenced by Nanjing Jiangbei New Area Biophamaceutical Public Service Platform Co., Ltd.

### Chromatin Immunoprecipitation (ChIP) and ChIP‐Seq

In brief, Chromatin immunoprecipitation was performed as follow**s**: intracellular protein‐DNA complexes were cross‐linked with 1% formaldehyde and stopped this process with glycine solution. The complexes were sonicated and subjected to chromatin‐conjugated IP using Anti‐Flag (F1804, Sigma‐Aldrich). After reversal of cross‐links, precipitated DNA was purified and analyzed by qPCR and Next‐Generation Sequencing, the ChIP‐seq databases were built by VAHTS ssDNA Library Prep Kit for Illumina (N620, Vazyme Biotech Co., Ltd), which were sequenced by Nanjing Jiangbei New Area Biophamaceutical Public Service Platform Co., Ltd.

Furthermore, alongside the recently acquired data presented in this paper, for ChIP‐seq analysis. We next map reads to genome by using the Bowtie2 v2.3.4.1 and short reads (length <35 bp) and low quality reads using Cutadapt v1.18. Next, it was converted into BigWig file by using the genomeCoverageBed and bedGraphToBigWig. The downstream analysis was performed by using MACS v2.1.2 and visualize them by using IGV2.8.13.

### Formaldehyde Crosslinking Cut‐Tag (FC‐Cut‐Tag) and Cut‐Tag Assays

To investigate alterations in the genomic distribution of WDR5 (13105, CST) following NSUN5 overexpression, FC‐cut‐tag assays were performed employing NovoNGS CUT&Tag 4.0 High‐Sensitivity Kit (for Illumina) (N259, Novoprotein, Shanghai, China), the FC‐cut‐tag databases were sequenced by Nanjing Jiangbei New Area Biophamaceutical Public Service Platform Co., Ltd.

Similarly, cut‐tag assays were employed by using Hyperactive Universal CUT&Tag Assay Kit for Illumina Pro (TD904, Vazyme Biotech Co., Ltd) to assess the alterations in the genome‐wide distribution of H3K4me1 (A22078, Abclonal), H3K4me2 (A22143, Abclonal) and H3K4me3 (A22146, Abclonal) pre‐ and post‐overexpression of NSUN5, and the cut‐tag databases were sequenced by Nanjing Jiangbei New Area Biophamaceutical Public Service Platform Co., Ltd.

### Purification of Recombinant Proteins

The pGEX‐6P‐1 vector was used to construct the expression plasmid of NSUN5 and the corresponding truncated body. The pET‐28a vector constructs the expression plasmid of WDR5, which was then transformed into *Escherichia coli* (E.coli) strain BL21 (DE3) and incubated with 0.1 mM. Isopropyl β‐D‐1‐thiogalactopyranoside (IPTG) (Beyotime, Shanghai, China) induces the expression of recombination proteins at 16 °C overnight. Glutathione S‐transferase‐tagged proteins (GST‐tag proteins) were purified using GSH Purose 4 Fast Flow (A41301, Qianchun Bio), while His‐tag proteins were purified using (A41002, Qianchun Bio), following the instructions. The purified proteins were immediately used for the experiments or frozen.

### Construction of Nsun5 Knockout Mouse Model

Guangzhou Cyagen Company was entrusted with the construction. The strategy was as follows: We used CRISPR/Cas‐mediated genome engineering to create a Nsun5 knockout Mouse model (C57BL/6N). The Cas9 protein and corresponding gRNA were transferred into the fertilized egg, and the Nsun5 gene was knocked out by targeting exons 3–6. Finally, the pups were genotyped by PCR followed by sequencing analysis.

Mice in the wild‐type and knockout gene groups were intraperitoneally injected DEN 2 mg kg^−1^ on the 14th day of birth, and CCl_4_ 5 µL g^−1^ (CCl_4_/ olive oil) ‐25:75) twice a week for 16 w after two weeks of administration. Mice were sacrificed during the 20th w of modeling. The Experimental Center approves the relevant animal ethical test certificates of Nantong University, and the animals are raised under animal welfare law.

### Animal Studies

Male BALB/c athymic nude mice (four‐week‐old) were obtained from the laboratory animal center of Nantong University and were bred under standard conditions (SPF levels) and cared for according to the institutional guidelines for animal care. All animal experiments were approved by the Institutional Animal Care and Use Committee of Nantong University (S20200330‐005). The 1 × 10^6^ cells in 100 µL of PBS were injected into the nude mice subcutaneously to establish the subcutaneous neoplasia mouse model. After six weeks, subcutaneous tumors were excised for immunohistochemistry (IHC). To establish the tail vein metastasis mice model, the 1 × 10^6^ cells in 100 µL of PBS were injected into the tail vein of nude mice. After six weeks, lung tissues were excised and subjected to hematoxylin and eosin (H&E) staining.

### Clinical Tissue Samples

Three cohorts of HCC tissue samples were collected at The Affiliated Taizhou People's Hospital of Nanjing Medical University. The first cohort included 24 HCC tissues and 24 matched non‐HCC tissues which were used for western blot assays. The second cohort included 12 HCC tissues and 12 matched non‐tumor tissues were used for Real‐time RT‐PCR. The last cohort included 90 HCC tissues and 90 adjacent non‐HCC tissues for Immunocytochemistry (IHC). The written informed consent was collected from the patients, and the experimental protocol got permission from the institutional ethics review board of The Affiliated Taizhou People's Hospital of Nanjing Medical University (2022‐008‐01). Pathologic analysis validated the identity of all specimens. Prior to surgery, no patients had chemotherapy or radiation.

### Statistical Analysis

Statistical significance of differences between groups was assessed using the GraphPad Prism7 software. Student's t‐test or one‐way ANOVA (analysis of variance) was applied to determine the significance between groups. The overall survival was estimated according to the Kaplan‐Meier method, and the log‐rank test determined significance. Image‐Pro Plus6 quantified the intensity of NSUN5 staining between p‐SMAD3, and their correlations were analyzed by Chi‐square test (Fisher's exact). Statistical significance was concluded at *p < 0.05, **p < 0.01, ***p < 0.001; ns: no statistical significance.

### Ethics Approval Statement and Patient Consent Statement

All animal experiments were approved by the Institutional Animal Care and Use Committee of Nantong University (S20200330‐005). The written informed consent was collected from the patients, and the experimental protocol got permission from the institutional ethics review board of The Affiliated Taizhou People's Hospital of Nanjing Medical University (2022‐008‐01)

## Conflict of Interest

The authors declare no conflict of interest.

## Supporting information



Supporting Information

## Data Availability

The data that support the findings of this study are available from the corresponding author upon reasonable request.
