# Supplementary material for: NSUN5 Facilitates Hepatocellular Carcinoma Progression by Increasing SMAD3 Expression
Source: Adv Sci (Weinh). 2024 Nov 12;12(2):2404083. doi: 10.1002/advs.202404083 (PMC11727281; doi:10.1002/advs.202404083)
Supplement: Supplementary file 1 — Supporting Information [file ADVS-12-2404083-s001.docx]

***NSUN5* Facilitates Hepatocellular Carcinoma Progression by Increasing SMAD3 Expression**

Hexu Han^1,#,*^,Chengcheng zhang^2,#^, Wenbo Shi^3,#^, Jiawei Wang^4, #^, Wenhui Zhao^5,#^, Yanping Du^1^, Zhibin Zhao^1^, Yifan Wang^1^, Maosong Lin^1^, Lei Qin^4^, Xiaoxue Zhao^2^_,_ Qianqian Yin^2^, Yiyi Liu^2^, Zhongqi Wang^2^, Jing Zhang^6^, Xiaomin You^6^, Guoxiong Zhou^6^, Honghui Wu^7^, Jun Ye^8^, Xianzhong He^9^, Weizhong Tian^10.*^, Hong Yu^11.*^ , Yin Yuan^12.*^, Qiang Wang^9,13,*^

1. Department of Gastroenterology, The Affiliated Taizhou People's Hospital of Nanjing Medical University, Taizhou School of Clinical Medicine, Nanjing Medical University, Taizhou, Jiangsu 225300, China.
2. Department of Medical Oncology, Longhua Hospital Affiliated to Shanghai University of Traditional Chinese Medicine, Shanghai University of Traditional Chinese Medicine, 725 Wanpingnan Road, Shanghai 200032, China.
3. Oncology major，Ruijin-Hainan Hospital, Shanghai Jiao Tong University School of Medicine, Hainan, China
4. Department of Hepatobiliary Surgery, The Affiliated Taizhou People's Hospital of Nanjing Medical University, Taizhou School of Clinical Medicine, Nanjing Medical University, Taizhou, Jiangsu 225300, People's Republic of China. Department of General Surgery, First Affiliated Hospital of Suzhou University, Suzhou, China
5. Department of Basic Medicine, Jiangsu College of Nursing, Huai’an, Jiangsu 223001, China.
6. Department of Gastroenterology, Affiliated Hospital of Nantong University, Nantong University, Jiangsu, 226001, China
7. Reproduction Medicine Centre, The Affiliated Taizhou People’s Hospital of Nanjing Medical University, Taizhou, China.
8. Center for Translational Medicine, The Affiliated Taizhou People's Hospital of Nanjing Medical University, Taizhou School of Clinical Medicine, Nanjing Medical University, Taizhou, Jiangsu 225300, China.
9. Department of Hepatobiliary Surgery, The First Affiliated Hospital of Anhui Medical University; Innovative Institute of Tumor Immunity and Medicine (ITIM); Anhui Provincial Innovation Institute for Pharmaceutical Basic Research; Anhui Province Key Laboratory of Tumor Immune Microenvironment and Immunotherapy, Hefei, Anhui, 230000, China.
10. Department of Radiology The Affiliated Taizhou People's Hospital of Nanjing Medical University, Taizhou School of Clinical Medicine, Nanjing Medical University, Taizhou, Jiangsu 225300, China.
11. Department of Pathology, The Affiliated Taizhou People's Hospital of Nanjing Medical University, Taizhou School of Clinical Medicine, Nanjing Medical University, Taizhou, Jiangsu 225300, China.
12. Department of Hepatobiliary Surgery, The Affiliated Taizhou People's Hospital of Nanjing Medical University, Taizhou School of Clinical Medicine, Nanjing Medical University, Taizhou, Jiangsu 225300, China.
13. Lead contact

**
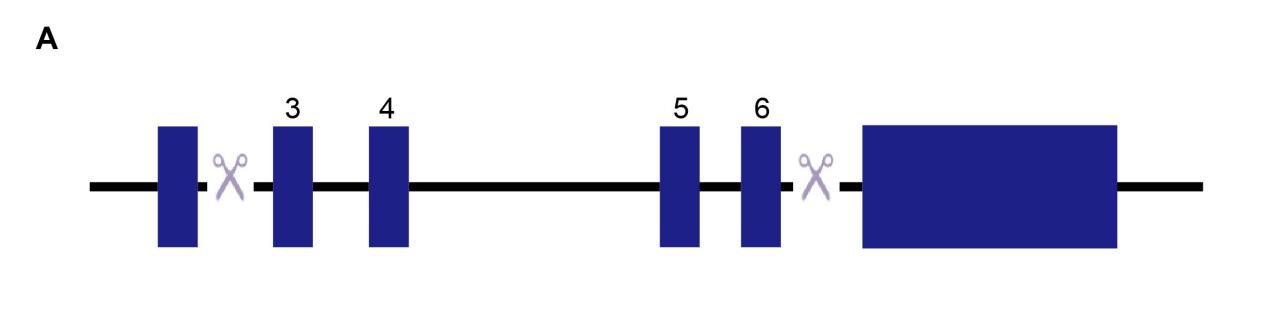
Figure S1. NSUN5 may be involved in mediating the malignant process of hepatocellular carcinoma**

1. Pattern diagram of construction strategies in Nsun5 knockout mice


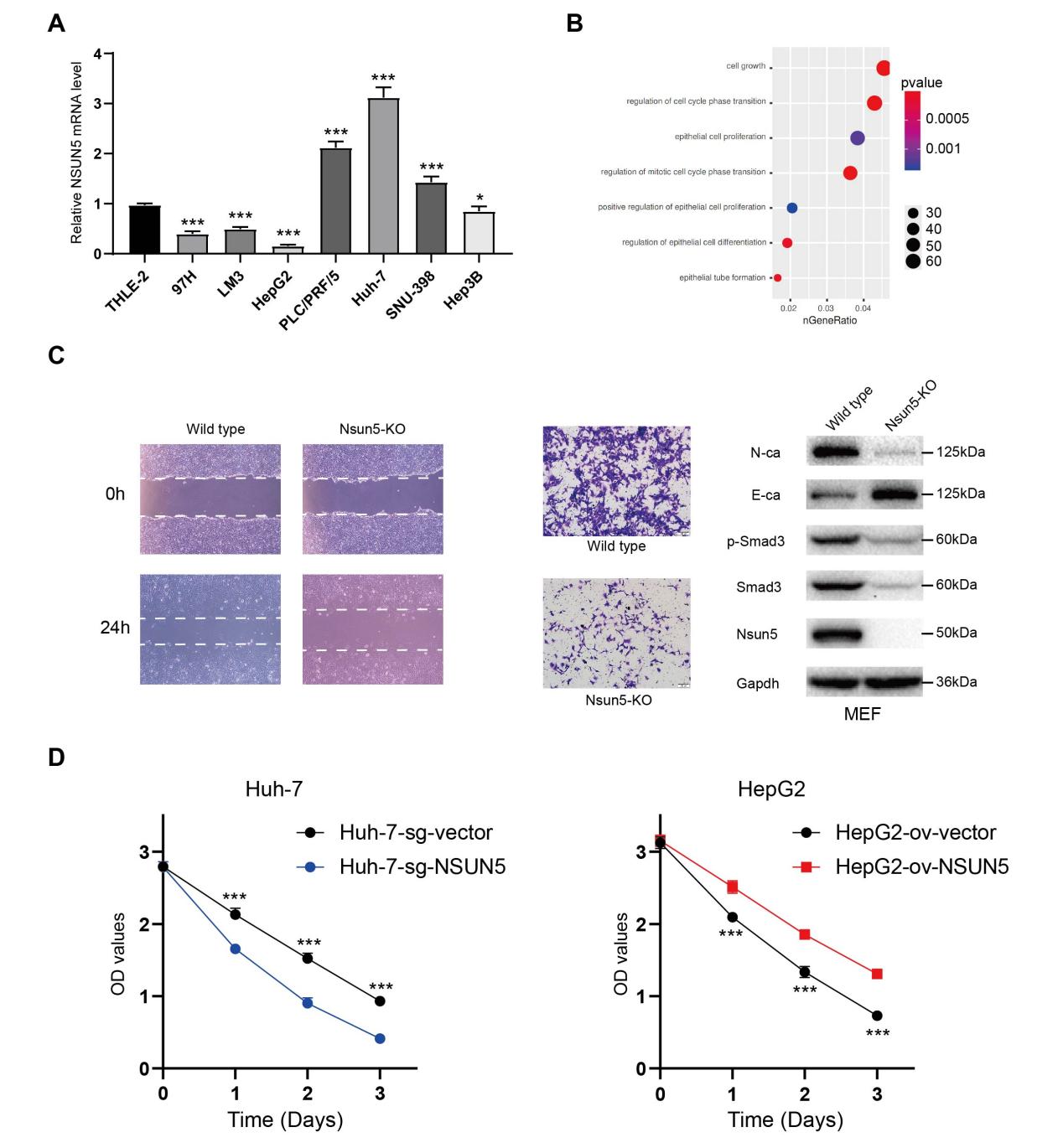


**Figure S2. Upregulation of NSUN5 induced EMT in HCC cells**

1. NSUN5 levels were evaluated in hepatocellular carcinoma cells via qPCR assays.
2. After overexpressing NSUN5, a GO enrichment analysis was performed on the differentially expressed genes and observed their involvement in biological processes (BP) related to the regulation of epithelial cells.
3. Comparison of mouse embryonic fibroblasts (MEFs) from wild-type and Nsun5 knockout mice showed that Nsun5 knockout could block epithelial-mesenchymal transformation.
4. The expression level of NSUN5 in HCC cells may modulate the cellular response to sorafenib. (n=3, p<0.0001)
